# Targeted modulation of thalamocortical and descending control networks in chronic shoulder pain: a longitudinal connectomics study

**DOI:** 10.3389/fpain.2026.1794217

**Published:** 2026-09-24

**Authors:** Li He, Wenjing Li, Lihang Gao, Xuan Du, Hongwei Ren, Qian Zhang, Yingchao Wu, Nicholas B. Dadario, Hongkai Chen, Michael E. Sughrue, Duanwei Wang, Hong Jin

**Affiliations:** 1 Department of Rehabilitation Medicine, Xichang Chuantou Health Technology Company Limited, Xichang, China; 2 Medical Department, Xijia Medical Technology Company Limited, Shenzhen, China; 3 Department of Neurosurgery, Columbia University Irving Medical Center, NY, New York, USA, United States; 4 Department of Psychiatry, The First Hospital of Hebei Medical University, The Mental Health Center of Hebei Medical University, The Mental Health Institute of Hebei Medical University, Shijiazhuang, Hebei, China

**Keywords:** chronic shoulder pain, connectomics, descending pain modulation, fMRI, functional connectivity, peripheral myofascial stimulation, pilot study, thalamocortical pathway

## Abstract

**Background:**

Central sensitization involves maladaptive plasticity in ascending nociceptive and descending modulatory pathways. This pilot study was designed with three integrated aims: (1) to characterize baseline thalamocortical and descending control network abnormalities in chronic shoulder pain (CSP) relative to healthy controls; (2) to examine whether these network features change following a standardized course of peripheral myofascial stimulation (PMS); and (3) to explore potential mechanistic pathways linking peripheral intervention to central network reconfiguration. While PMS has been reported anecdotally as effective for musculoskeletal pain, preliminary empirical evidence from randomized controlled trials remains limited, and its central neural mechanisms remain unclear.

**Methods:**

Twelve patients with CSP underwent longitudinal resting-state fMRI before and after a 10-session PMS intervention. A matched healthy control cohort (*n* = 18) provided cross-sectional baseline normative data for disease characterization only; no longitudinal data were collected from controls. High-resolution parcellation (HCP-MMP1.0) was used to quantify connectivity in *a priori* hypothesized networks. Statistical analysis employed a tiered approach combining whole-brain Network-Based Statistics (*N*BS) and hypothesis-driven Region of Interest (ROI) analyses, rigorously controlled for site effects using ComBat harmonization.

**Results:**

At baseline, patients exhibited trend-level reduced thalamo-somatosensory coupling (Cohen's d = 0.72, *p* = 0.054) compared to controls, which correlated with disease burden. Following treatment, we observed: (1) a downregulation of ACC–brainstem connectivity at the ROI level (Pre 0.017 ± 0.209, Post −0.149 ± 0.211, *Δ* = −0.166; Wilcoxon *p* = 0.012, d = −0.82), which did not survive Bonferroni correction across the eight *a priori* edges (corrected *p* = 0.098); and (2) patient-specific change in thalamo-somatosensory connectivity, which was associated with change in present pain intensity (*Δ*PPI, *ρ* = −0.73). Control analyses in visual and limbic networks were null; this is compatible with, but does not confirm, anatomical restriction.

**Conclusions:**

PMS is associated with the reconfiguration of specific bottom-up gating and top-down control circuits in CSP. While the absence of a sham control and the small sample size (*n* = 12) limit causal inference and generalizability, the large effect sizes and high anatomical specificity suggest these are preliminary biological signals worthy of further investigation. These preliminary findings provide mechanistic targets for future adequately powered randomized controlled trials.

## 1 Introduction

Chronic shoulder pain (CSP) represents a debilitating musculoskeletal condition that affects a significant proportion of the adult population, contributing substantially to the global burden of disease and disability (1, 2). While often initiated by peripheral pathology, such as rotator cuff tendinopathy or adhesive capsulitis (3), the persistence of pain frequently becomes uncoupled from the initial tissue injury. This transition from acute to chronic pain is thought to be driven by maladaptive neuroplasticity within the central nervous system, a phenomenon broadly termed “central sensitization” (4, 5). Central sensitization involves a state of neuronal hyperexcitability characterized by amplified synaptic transmission, reduced activation thresholds, and expanded receptive fields in spinal and supraspinal nociceptive pathways. Key mechanisms include increased glutamatergic signaling, reduced GABAergic and glycinergic inhibition, and structural reorganization of synaptic connections in the dorsal horn and higher-order pain-processing regions (4). Despite this understanding, current therapeutic strategies for CSP remain peripherally focused, yielding inconsistent outcomes and offering minimal insight into whether effective treatments are affecting specific central circuits or just minimizing symptoms. A critical translational gap therefore remains in both a refined central understanding of the neuroanatomic substrates involved in CSP and also how peripheral therapeutic inputs can effectively reverse these entrenched central maladaptations.

The neuroanatomic architecture of chronic pain involves a distributed network governing sensory discrimination, affective evaluation, and descending modulation (6). Two primary theoretical frameworks dominate our understanding of this circuitry. First, the classic Gate Control Theory posits that non-nociceptive afferent input can modulate nociceptive transmission at the spinal and supraspinal levels, effectively “closing the gate” to pain signals (7, 8). The thalamus, as the central relay station, acts as the critical “supraspinal gatekeeper,” regulating the flow of sensory information to the somatosensory cortex (S1/S2) (9). Disruption in thalamocortical functional connectivity has been repeatedly implicated in chronic pain states, often manifesting as thalamocortical dysrhythmia (10). Second, the Descending Inhibitory System, anchored by the anterior cingulate cortex (ACC) and its projections to the brainstem (e.g., periaqueductal gray and rostral ventromedial medulla), exerts top-down control over spinal nociception (11). Dysfunction in this top-down regulation-often characterized by a shift from inhibition to facilitation-is a hallmark of central sensitization (12, 13).

Peripheral Myofascial Stimulation (PMS) is a manual therapy technique involving targeted mechanical stimulation of myofascial trigger points and hypertonic muscular regions. While PMS has been anecdotally reported as clinically effective for musculoskeletal pain in some observational studies and clinical practice guidelines, preliminary empirical evidence from large-scale randomized controlled trials remains limited (14, 15). Furthermore, its central neural mechanisms remain elusive. Specifically, it remains unclear whether PMS exerts its analgesic effects by re-engaging bottom-up thalamocortical sensory gating, resynchronizing top-down descending modulatory control, or some version of both of these processes. Prior neuroimaging studies have been limited by cross-sectional designs and coarse anatomical atlases that lack the anatomical granularity necessary to dissociate the subregional somatosensory and modulatory connectomic networks (16). Critically, few studies have employed longitudinal within-subjects designs capable of capturing the dynamic reconfiguration of these specific circuits due to successful treatment over time.

To address these gaps, this high-resolution longitudinal pilot study was designed with three integrated aims: (1) to characterize baseline abnormalities in thalamocortical and descending control networks in CSP patients relative to healthy controls; (2) to examine whether these network features change following a standardized course of PMS; and (3) to explore potential mechanistic pathways linking peripheral intervention to central network reconfiguration. We leveraged the high-granularity HCP-MMP1.0 parcellation to dissect specific connectivity changes in *a priori* networks which have been highly implicated in the literature. We therefore formulated two mechanistic hypotheses: (1) CSP is characterized by a baseline “opening” or disruption of the thalamocortical sensory gate; and (2) effective PMS induces anatomically specific reconfiguration of bottom-up thalamocortical pathways and/or top-down ACC-brainstem control, rather than a diffuse global network alteration.

## 2 Materials and methods

### 2.1 Participants and study design

A prospective cohort of 21 patients with CSP was recruited from the Department of Rehabilitation at Chuantou Xichang Hospital between August 2024 and July 2025. CSP was defined as chronic shoulder pain persisting for > 3 months accompanied by restricted active range of motion (ROM). Inclusion required predominantly unilateral pain, or bilateral pain with a clearly designated primary affected side. One of the 12 analysed patients had bilateral involvement (Table 1) and was retained as a protocol deviation; clinical ratings used the more painful side. The research has been approved by the ethics committee of Xichang Chuantou Health Technology Company Limited (ethics number: 202304).

**Table 1:** Clinical demographics.

Characteristic (mean ± SD)	CSP Patients (*n* = 12)	HC (*n* = 18)
Demographics		
*Age (years)*	55.9 ± 7.6	56.2 ± 8.1
*Age range*	36–64	36–65
*Female, n (%)*	8 (67%)	11 (61%)
*Male, n (%)*	4 (33%)	7 (39%)
Affected Shoulder		
*Right, n (%)*	8 (67%)	-
*Left, n (%)*	3 (25%)	-
*Bilateral, n (%)*	1 (8%)	-
VAS (0–10)	4.5 ± 2.2	
*PPI (0–5)*	3.1 ± 0.3	-
*SF-MPQ PRI-T (sensory + affective, 0–45)*	8.1 ± 3.0	-
*Sensory (0–33)*	4.5 ± 2.2	-
*Affective (0–12)*	3.6 ± 1.2	-
*Affective subscore*	3.6 ± 1.2	-
Function		
*Shoulder flexion (°)*	124 ± 20	-
*Shoulder abduction (°)*	128 ± 35	-
*MBI (0–100)*	99.3 ± 1.3	-
Comorbidities		
*HAMA (anxiety)*	13.4 ± 8.4	-
*HAMD (depression)*	5.2 ± 4.9	-
*PSQI (sleep)*	10.9 ± 4.7	-

One participant presented with bilateral symptoms; the more painful shoulder was designated as the affected side for analysis.

#### 2.1.1 Clinical phenotyping approach

CSP encompasses heterogeneous pathoanatomical subtypes (e.g., rotator cuff tendinopathy, adhesive capsulitis). In this pilot study, we adopted an inclusive phenotypic approach, enrolling patients meeting IASP chronic pain criteria regardless of specific structural diagnosis. This rationale was twofold: (1) Pragmatic generalizability: Real-world CSP patients often present with multi-factorial pathology; and (2) Central mechanism hypothesis: We hypothesized that PMS targets central neuroplastic changes (e.g., thalamocortical gating) shared across structural subtypes, analogous to central sensitization mechanisms observed across diverse chronic pain conditions. While this approach maximizes translational relevance, it limits the ability to attribute effects to specific peripheral lesions.

#### 2.1.2 Inclusion and exclusion criteria

##### 2.1.2.1 Inclusion criteria were

(1) Chronic shoulder pain fulfilling the International Association for the Study of Pain (IASP) definition of chronic pain (17), predominantly unilateral or, if bilateral, with a designated primary affected side; (2) Self-reported limitation of active ROM compared with the contralateral side; (3) Naïve to shoulder surgery on the affected side; and (4) Consent to receive the standardized PMS protocol independent of specific structural sub-typing. The overall experimental design and timeline are illustrated in Figure 1A.

**Figure 1:**
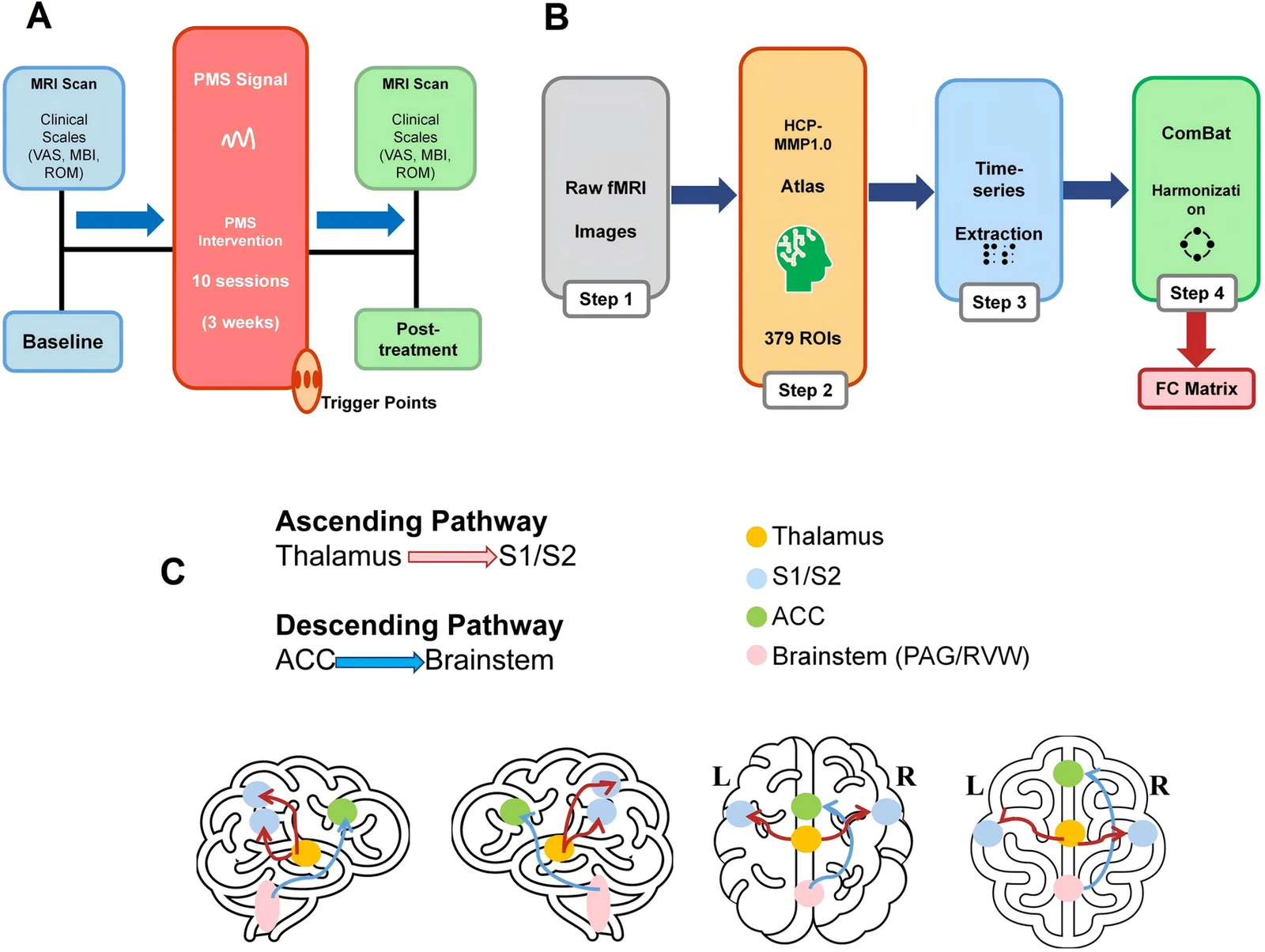
Experimental design and analytical framework. **(A)** Longitudinal Protocol; **(B)** Analysis Pipeline; **(C)** Regions of Interest (ROIs).

##### 2.1.2.2 Exclusion criteria included

(1) History of comorbid neurological or psychiatric disorders; (2) Substance abuse; (3) Contraindications for MRI scanning (e.g., metallic implants, claustrophobia); (4) Gross structural brain abnormalities detected during the initial MRI; or (5) Withdrawal of consent.

##### 2.1.2.3 Final sample and attrition

Following rigorous screening, 9 participants were excluded due to follow-up attrition (*n* = 6), withdrawal of consent (*n* = 2), or MRI intolerance (*n* = 1). Consequently, 12 patients with complete, high-quality longitudinal MRI data were included in the final analyses. As a sensitivity analysis for the one bilateral case, ACC–brainstem *Δ*FC remained negative after excluding that participant (*n* = 11; Wilcoxon *p* = 0.024).

#### 2.1.3 Sample size considerations

Given the exploratory nature of this pilot study and the absence of prior effect size estimates for PMS-induced connectivity changes in CSP, a formal *a priori* power calculation was not feasible. The sample size of 12 patients was determined by practical constraints including recruitment feasibility, MRI scan availability, and the exploratory scope of the study. We acknowledge that this sample size is insufficient to detect small-to-moderate effects and that the study is underpowered for definitive conclusions. All findings should be interpreted as hypothesis-generating, with effect size estimates intended to inform sample size calculations for future confirmatory trials rather than as precise population parameters.

#### 2.1.4 Clinical assessment

All evaluations were performed by a single assessor blinded to imaging data at baseline and post-treatment. (1) Pain. Current intensity was rated on a Visual Analog Scale (VAS; 0–10 cm). The Present Pain Intensity (PPI; 0–5) and Short-Form McGill Pain Questionnaire (SF-MPQ; 0–45) were used to assess sensory (0–33) and affective (0–12) pain qualities; (2)Function. Active shoulder flexion and abduction were measured with a standard goniometer. The Modified Barthel Index (MBI; 0–100) assessed activities of daily living; (3)Psychological and Sleep. The Hamilton Anxiety Scale (HAMA; 0–56), Hamilton Depression Scale (HAMD-17; 0–52), and Pittsburgh Sleep Quality Index (PSQI; 0–21) evaluated anxiety, depression, and sleep quality, respectively.

### 2.2 Healthy control (HC) cohort

To establish a cross-sectional normative neuroimaging baseline for disease characterization, a Healthy Control (HC) cohort was curated from the OpenNeuro dataset “Brain connectivity predicts placebo response across chronic pain clinical trials” (18). Rigorous selection criteria were applied to ensure comparability: (1) Pain-free status: Only healthy participants were retained; (2) Scanner consistency: Only datasets acquired on Siemens MRI systems were selected to match the CSP cohort manufacturer; (3) Demographic matching: Participants aged < 35 years were excluded to align with the CSP age profile. The final HC sample comprised 18 age- and sex-matched participants. There were no significant between-group differences in age (*p* > 0.05) or sex distribution (*χ*^2^ test, *p* > 0.05). Demographic and clinical characteristics are summarized in Table 1.

We acknowledge that the HC cohort (*n* = 18) serves solely as a cross-sectional baseline reference for disease characterization and does not function as a control for the intervention. Specifically, HC participants were not assessed longitudinally and did not undergo the PMS treatment. Therefore, the key longitudinal findings rely on pre-post comparisons within the single patient group, which limits causal inference. The observed changes in functional connectivity could reflect non-specific effects (e.g., expectancy or placebo effects), repeated scanning, or natural recovery, rather than the specific effects of PMS. Future adequately powered randomized controlled trials with sham control groups are required to establish causal relationships.

### 2.3 Intervention

All participants received 10 sessions of Peripheral Myofascial Stimulation (PMS) over a period of 3–4 weeks. Treatments were administered by a single licensed practitioner with > 10 years of experience to ensure protocol consistency.

The standardized PMS protocol was adapted from established myofascial trigger point therapy principles (19, 20) and consisted of the following components: (1) Pre-treatment assessment: Each session began with palpation of the affected shoulder girdle to identify hypertonic myofascial trigger points (MTrPs) and regions of restricted fascial mobility; (2) Targeted stimulation: Using a sterile, single-use solid filament needle (0.25 mm diameter, 40 mm length), the practitioner applied precise mechanical stimulation to identified MTrPs, with depth adjusted according to anatomical location (subcutaneous to intramuscular, avoiding neurovascular structures); (3) Stimulation parameters: Each MTrP received 3–5 insertions with gentle rotational manipulation, held for 10–15 s per insertion, with local twitch response elicitation when possible; (4) Session duration: Each session lasted 20–30 min, targeting 3–5 MTrPs per session; (5) Post-treatment: Participants were advised to avoid vigorous shoulder activity for 24 h post-session. The practitioner documented MTrP locations, insertion depths, and patient-reported pain responses (0–10 scale) for each session to ensure protocol fidelity.

Raw fMRI data were processed using a high-resolution connectomics approach. Time-series were extracted using the HCP-MMP1.0 multi-modal atlas (379 regions), followed by ComBat harmonization to correct for multi-site scanner effects before network construction. Anatomical visualization of the *a priori* hypothesized network nodes within the ascending sensory (Thalamus, S1, S2) and descending modulatory (ACC, Brainstem) pathways.

### 2.4 Data acquisition

CSP Cohort: High-resolution T1-weighted anatomical images were acquired on a Siemens MAGNETOM Prisma 3.0 T scanner (Siemens Healthineers, Erlangen, Germany) using a 3D MPRAGE sequence (voxel size=0.94 × 0.94 × 0.94 mm^3^; TR = 1900ms; TE = 4.28 ms; flip angle=9°; 192 sagittal slices). Resting-state functional images were acquired using a 2D gradient-echo EPI sequence (voxel size=3 × 3 × 3 mm^3^; TR = 3270 ms; TE = 15 ms; 55 axial slices; flip angle=90°; 200 volumes; acquisition time=10 min 54 s). Participants were instructed to keep their eyes closed, remain awake, and refrain from mind-wandering. A foam pad was used to minimize head motion. HC Cohort: To minimize hardware-related variability, HC datasets were selected exclusively from Siemens 3.0 T scanners. Acquisition protocols were broadly compatible: T1-weighted (MPRAGE, TR = 2500 ms, TE = 3.36 ms, voxel size=1.0 × 1.0 × 1.0 mm^3^) and rs-fMRI data (EPI, TR = 2500 ms, TE = 30 ms, voxel size=3.0 × 3.0 × 3.0 mm^3^, 200 volumes).

### 2.5 Preprocessing and parcellation

Resting-state fMRI data were preprocessed using a standard pipeline implemented in FMRIB Software Library (FSL, version 6.0.5; https://fsl.fmrib.ox.ac.uk/fsl/fslwiki) (21) and AFNI (version 23.0.01; https://afni.nimh.nih.gov) (22), including: (1) rigid-body realignment to the middle volume using MCFLIRT; (2) slice timing correction using Fourier interpolation; (3) censoring of high-motion volumes (DVARS > 2 SD) (21); (4) co-registration of functional images to individual T1-weighted anatomical images using boundary-based registration (BBR); and (5) spatial normalization to MNI152 standard space (2 mm isotropic) using non-linear diffeomorphic warping (FNIRT) (23, 24). T1-weighted images were skull-stripped using BET and segmented into gray matter, white matter, and cerebrospinal fluid using FAST. Physiological and motion artifacts were mitigated using the CompCor approach (25), with 5 principal components extracted from white matter and cerebrospinal fluid masks; global signal regression was not applied to avoid introducing spurious anti-correlations (26). The complete neuroimaging analysis pipeline is summarized in Figure 1B.

Whole-brain parcellation employed the HCP-MMP1.0 multimodal atlas, yielding 379 ROIs (360 cortical regions and 19 additional subcortical regions) (16). This high granularity enabled the critical dissociation of somatosensory subregions, specifically distinguishing S1 (areas 1, 2, 3a, 3b) from S2 opercular fields (op1–4). Consistent with *a priori* hypotheses regarding nociceptive processing (27, 28), analyses focused on nodes encompassing the thalamus, S1, S2, ACC, insula, amygdala, PFC, and brainstem (Figure 1C).

### 2.6 Cross-site harmonization and validation

To rigorously address residual scanner- and protocol-related effects, ComBat harmonization was applied to the functional connectivity matrices (29). Scanning site Site A [Xichang Chuantou Health Technology Company Limited] vs. Site B [OpenNeuro contributing sites]] was modeled as the batch variable, with age and sex included as biological covariates to preserve meaningful variance. To specifically rule out potential confounds arising from the TR discrepancy (2500 ms vs. 3270 ms), we conducted a simulation-based validation analysis. This confirmed that TR heterogeneity introduced negligible deviation in functional connectivity estimates (mean RMSE=0.0034). Furthermore, Principal Component Analysis (PCA) demonstrated effective attenuation of site-related clustering. Importantly, primary treatment effects were evaluated using within-subject longitudinal comparisons within the CSP cohort, rendering the main findings largely independent of cross-site technical factors.

### 2.7 Statistical analysis

Functional connectivity within *a priori* defined ascending sensory-discriminative and descending modulatory pathways was examined. Pre- to post-treatment changes in connectivity were evaluated using two-tailed paired t-tests for normally distributed variables or Wilcoxon signed-rank tests for non-normally distributed variables, as determined by Shapiro–Wilk tests of normality (*α* = 0.05). The choice between parametric and non-parametric tests was made *a priori* based on distributional properties of the connectivity data. Shapiro–Wilk tests were applied to each of the eight *Δ*FC variables: seven edges did not deviate from normality (*p* > 0.05) and were tested with paired t-tests; ACC–brainstem *Δ*FC departed from normality (Shapiro–Wilk *p* = 0.026) and was tested with the Wilcoxon signed-rank test (*p* = 0.012; paired t *p* = 0.017 for comparison). The baseline patient–control comparison was assessed using an independent t-test after confirming normality in both groups. For behavioral correlations, Spearman's rank correlation was chosen *a priori* to accommodate potential non-normality in clinical change scores given the small sample size. These test selections were determined before data analysis and were not altered *post hoc*.

To ensure full transparency in multiple-comparison control, we defined three distinct testing families *a priori*:

Family 1: Whole-brain exploratory analysis. NBS was applied across all 71,631 possible edges with FWE correction (5,000 permutations, initial cluster-forming threshold *p* < 0.001) to identify any treatment-associated connected components.
Family 2: Hypothesis-driven ROI analyses. We tested eight *a priori* edges using paired t-tests. These edges were fully pre-specified before data analysis based on established neuroanatomical literature on pain processing (6, 9, 11, 27, 28). Given the exploratory pilot design, uncorrected *p* values are reported as primary results, accompanied by Cohen's d. For completeness, Bonferroni-corrected thresholds (*α* = 0.0063 for 8 comparisons) are reported as a sensitivity analysis.
Family 3: Behavioral correlations. Associations between connectivity changes (*Δ*FC) and clinical changes (*Δ*VAS, *Δ*PPI, *Δ*MBI) were assessed using Spearman's rank correlation. Resulting *p* values were adjusted via the FDR procedure (30) across all 24 tested correlations (8 edges×3 clinical outcomes). Raw uncorrected *p* values are also reported in Table 2 for transparency.

**Table 2:** Treatment-Associated changes in pain-related network connectivity.

Pathway	Network	Pre-treatment	Post-treatment	Healthy Control	*Δ* FC	*Δ* %	*p*-value	Cohen's d	Clinical correlation
*A PRIORI* HYPOTHESIZED PATHWAYS									
Thalamus–S1/S2	Ascending sensory	−0.126 ± 0.097	−0.117 ± 0.091	−0.039 ± 0.121	0.009	7.14%	0.18	0.42	*Δ*PPI: *ρ*=−0.73, *p* = 0.007
ACC–Brainstem	Descending modulatory	0.017 ± 0.209	−0.149 ± 0.211	−0.125 ± 0.202	−0.166	—	0.012 (Wilcoxon)	−0.82	*Δ*PPI: ρ = + 0.67, *p* = 0.016 (uncorrected)
CONTROL PATHWAYS (Null Results)									
Thalamus–Limbic	Affective	0.380 ± 0.190	0.360 ± 0.200	—	−0.020	−5.3%	0.42	−0.10	*Δ*PPI: ρ=−0.23, *p* = 0.467
Visual system	Non-pain control	—	—	—	—	—	0.621	0.15	n.s. (ρ < 0.15)
Default mode	Non-pain control	—	—	—	—	—	0.58	−0.16	n.s. (ρ < 0.16)

Data are presented as mean ± standard deviation. *Δ*FC represents the absolute difference (Post−Pre), while *Δ*% represents the relative percentage change. *P*-values for the primary pathway comparisons are uncorrected. The ACC–Brainstem comparison was one of eight *a priori* ROI edges tested; it was the only edge with an uncorrected *p* < 0.05 and did not survive Bonferroni correction for multiple comparisons (*α* = 0.0063, 8 edges; corrected *p* = 0.098). Complete results for all eight prespecified edges (test statistics, raw and Bonferroni-adjusted *p*-values, and effect sizes) are provided in Supplementary Table 1. We therefore report the uncorrected *p*-value as the primary result in this exploratory pilot design, with the corrected threshold presented as a sensitivity analysis. Clinical correlations were assessed using Spearman's ρ with FDR correction across 24 tests (8 edges×3 clinical outcomes); raw uncorrected *p*-values for pathway comparisons and FDR-corrected *p*-values for clinical correlations are reported.

To balance the risks of Type I and Type II error in this pilot investigation, a tiered statistical approach was implemented:

#### 2.7.1 Whole-brain exploration

Network-Based Statistic (NBS) was applied with strict Family-Wise Error (FWE) correction (5,000 permutations, initial cluster-forming threshold *p* < 0.001) (31) to identify any treatment-associated connected components across the whole brain. This analysis served as a control for global, non-specific network changes.

#### 2.7.2 Hypothesis-driven ROI analysis

For the purpose of multiple comparison correction, we defined our primary hypothesis-driven ROI analysis as a single “testing family” consisting of the following eight *a priori* edges, which were fully pre-specified based on our mechanistic hypotheses and prior literature (6, 9, 11, 27, 28):

(1) Thalamus-S1 (areas 1, 2, 3a, 3b): Ascending discriminative somatosensory pathway;
(2) Thalamus-S2 (op1-op4): Ascending integrative somatosensory pathway;
(3) Thalamus-insula: Affective-motivational pathway;
(4) Thalamus-amygdala: Emotional-evaluative pathway;
(5) ACC-brainstem (periaqueductal gray, rostral ventromedial medulla): Descending modulatory pathway;
(6) ACC-thalamus: Cortico-thalamic feedback pathway;
(7) ACC-prefrontal cortex (areas 9, 10, 46): Cognitive-evaluative pathway;
(8) S1-S2: Intracortical somatosensory integration pathway.

For each edge, functional connectivity was computed as the Fisher z-transformed Pearson correlation coefficient between the average time series of the constituent ROIs. Pre- to post-treatment changes were assessed using paired t-tests, except for ACC–brainstem (Wilcoxon signed-rank test, per the Shapiro–Wilk screening above). Given the pre-specified, hypothesis-driven nature of these analyses and the exploratory pilot design, uncorrected *p*-values are reported for these specific connections, accompanied by Cohen's d to indicate effect size and practical significance. We acknowledge that uncorrected *p*-values increase the risk of Type I error; therefore, these findings are interpreted as hypothesis-generating rather than confirmatory. For completeness, we also report Bonferroni-corrected thresholds (*α* = 0.0063 for 8 comparisons) as a sensitivity analysis.

#### 2.7.3 Behavioral correlations

Associations between changes in functional connectivity (*Δ*FC = Post−Pre) and clinical outcome changes (*Δ*Clinical = Post−Pre) were assessed using Spearman's rank correlation to accommodate potential non-normality in the small sample. Resulting *p*-values were adjusted for multiple comparisons via the False Discovery Rate (FDR) procedure (30) across all tested connectivity-behavior correlations (*n* = 8 edges×3 primary clinical outcomes=24 tests). Raw (uncorrected) *p*-values are also reported for transparency.

All statistical analyses were performed in Python 3.11 utilizing the pandas (version 2.0.3) and SciPy (version 1.11.2) libraries (32). Effect sizes were computed using the effsize package (version 0.8.1).

## 3 Results

### 3.1 A quantitative signature of chronic shoulder pain

At baseline, thalamocortical coupling exhibited a trend-level reduction compared to healthy controls (two-tailed independent t-test: *p* = 0.054, Cohen's d = 0.72, Figure 2A). This does not reach conventional statistical significance (*α* = 0.05) and therefore cannot be interpreted as a definitive disease signature; rather, the medium-to-large effect size suggests a biologically plausible signal that was likely underpowered in this small sample. We caution that this trend-level finding requires confirmation in larger samples. Importantly, this connectivity metric exhibited preliminary associations with multiple independent clinical indices at the individual level (Figures 2B–D). This metric scaled with individual disease burden: lower thalamo-somatosensory connectivity was associated with higher spontaneous pain (Visual Analog Scale: VAS, *ρ* = −0.68, *p* = 0.003), reduced active range of motion (*ρ* = 0.63, *p* = 0.009), and greater disability in activities of daily living (Modified Barthel Index: MBI, *ρ* = 0.58, *p* = 0.020; Figures 2B–D). These convergent associations indicate that thalamo-S1/S2 connectivity captures inter-individual variability in clinical severity in CSP, supporting its potential relevance as a disease biomarker.

**Figure 2:**
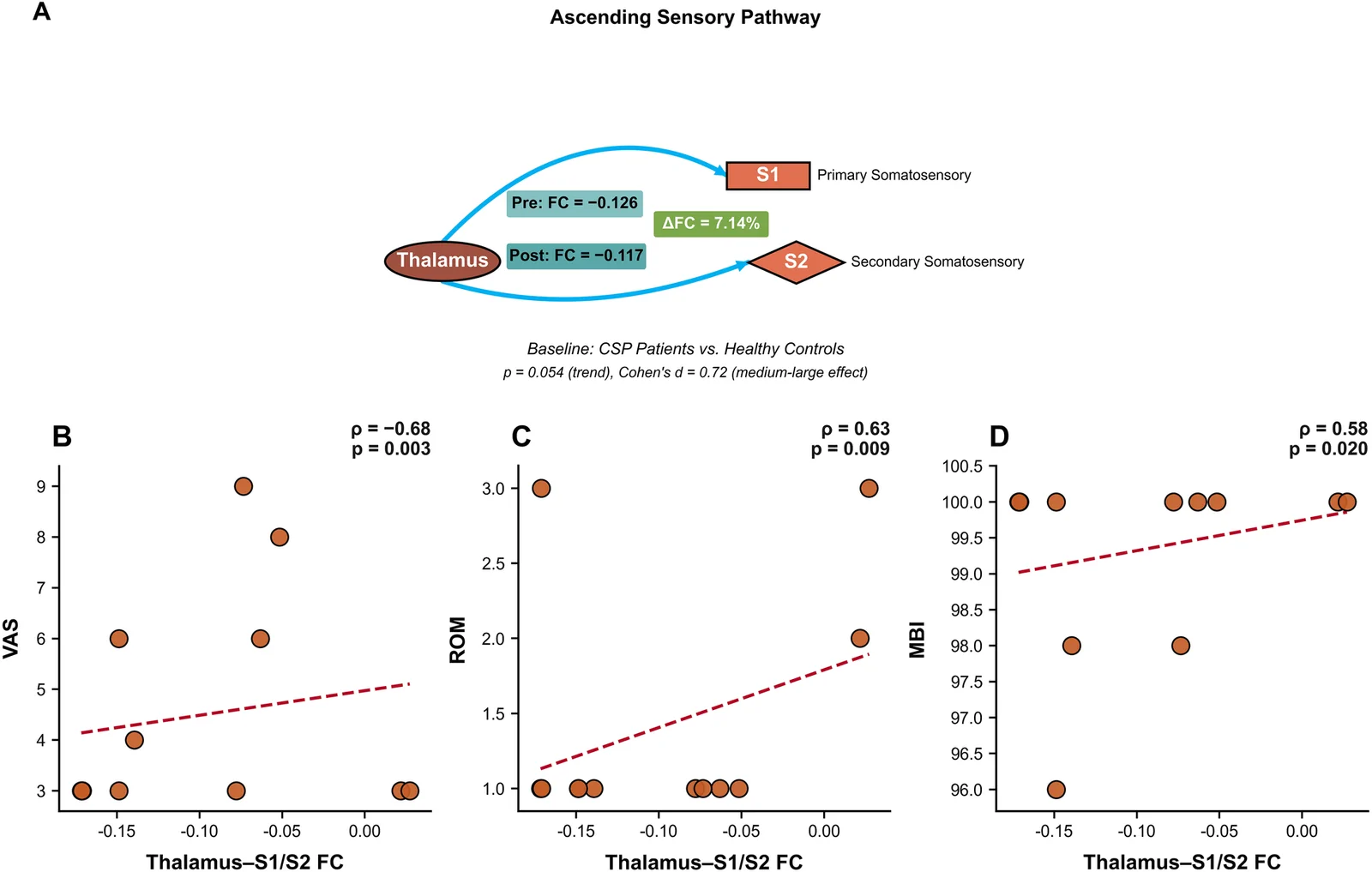
Baseline thalamocortical dysconnectivity as a potential neural correlate of clinical severity. **(A)** Schematic representation of the ascending sensory pathway; **(B–D)** Scatter plots demonstrating correlations between baseline thalamocortical connectivity and individual clinical metrics.

### 3.2 Mechanism I: Top-down inhibitory reset

Following the 10-session intervention, ACC–brainstem ROI connectivity decreased in patients (Pre 0.017 ± 0.209, Post −0.149 ± 0.211, *Δ* = −0.166; Wilcoxon signed-rank *p* = 0.012, Cohen's d = −0.82; Table 2 and Figures 3A,B). Treatment was associated with a selective change in descending control circuitry. This comparison was one of eight *a priori* ROI edges tested; it did not survive Bonferroni correction for multiple comparisons (*α* = 0.0063, 8 edges; corrected *p* = 0.098). We therefore report the uncorrected *p* value as the primary result in this exploratory pilot design, with the corrected threshold presented as a sensitivity analysis. Across the pre-specified Family 3 outcomes, ACC–brainstem change was not significantly associated with *Δ*VAS (*ρ* =  + 0.36, *p* = 0.27, *n* = 11) or *Δ*MBI (*ρ* = −0.25, *p* = 0.44); its uncorrected association with *Δ*PPI (*ρ* =  + 0.67, *p* = 0.016) is reported in Table 2.

**Figure 3:**
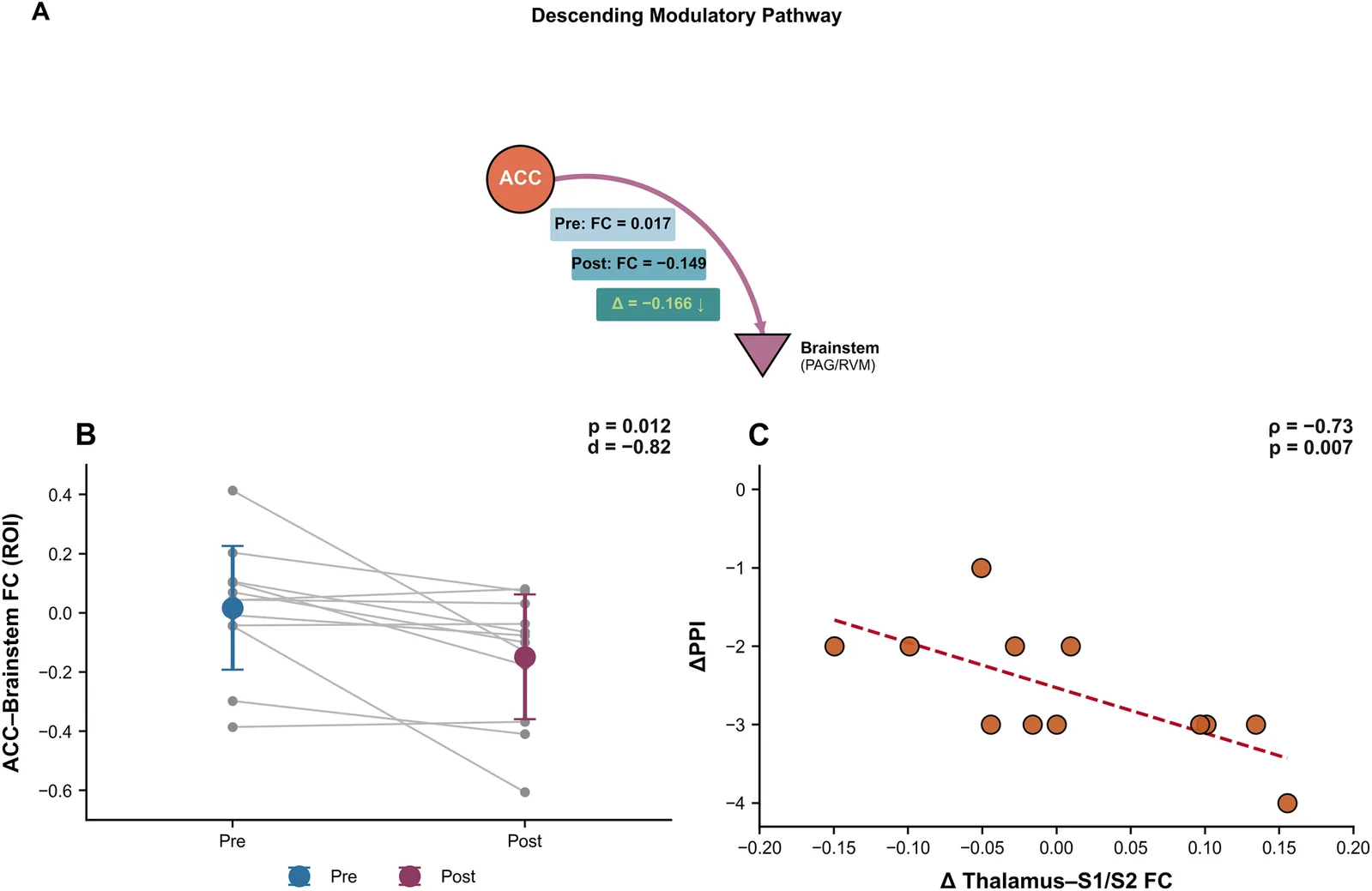
Descending control and ascending sensory circuits following treatment. **(A)** Schematic of the ACC–brainstem descending modulatory pathway; **(B)** Individual patient ACC–brainstem ROI connectivity before and after treatment (group means: Pre 0.017 ± 0.209, Post −0.149 ± 0.211; Wilcoxon *p* = 0.012, d = −0.82; Bonferroni-corrected *p* = 0.098 across eight *a priori* edges); **(C)** Association between individual thalamo–S1/S2 connectivity change and change in present pain intensity (*Δ*PPI; Spearman *ρ* = −0.73, *p* = 0.007). All findings were exploratory.

### 3.3 Mechanism II: bottom-up sensory gate reconfiguration

At the group level, thalamo-S1/S2 connectivity showed no significant change following treatment (+6%, paired t-test: *p* = 0.18, d = 0.42; Table 2). This null result at the group level does not support a “restoration” mechanism. However, individual variability in pathway plasticity was strongly related to clinical outcome. Patient-specific changes in thalamo-somatosensory connectivity correlated negatively with changes in present pain intensity (*Δ*PPI, Spearman's *ρ* = −0.73, *p* = 0.007; Table 2 and Figure 3C). No corresponding associations were observed for thalamo-limbic, cerebellar, or visual connections (all |*ρ*| < 0.22, all *p* > 0.35), indicating anatomical specificity of this correlation. We interpret this pattern cautiously: while the group-level change was not significant, the strong individual-level correlation suggests that thalamo-somatosensory plasticity may be a relevant mechanistic target in a subset of responders, warranting investigation in larger samples.

### 3.4 Anatomical and statistical specificity

Whole-brain permutation testing (NBS; FWE-corrected) revealed no significant pre- to post-treatment connected components at the specified statistical threshold. This indicates that no evidence for widespread network change was detected; it does not confirm that the treatment effects were anatomically restricted (Table 2). Direct comparison of the thalamo-limbic affective axis also yielded a null result (d = −0.10, *p* = 0.42). Similarly, no significant changes were observed in the visual system (d = 0.15, *p* = 0.621), default mode network (d = −0.16, *p* = 0.580), or sensorimotor network (d = 0.08, *p* = 0.782). Critically, clinical correlations were equally specific: treatment-associated changes in pain outcomes (*Δ*PPI, *Δ*VAS) showed no corresponding associations with thalamo-limbic (*ρ* = −0.23, *p* = 0.467), visual, or default mode connectivity changes (all |*ρ*| < 0.30, all *p* > 0.35). Together, these control analyses are compatible with, but do not confirm, restriction of treatment-related connectivity changes to sensory-discriminative (thalamo-S1/S2) and descending modulatory (ACC-brainstem) circuits rather than diffuse or global network alterations.

## 4 Discussion

Through longitudinal, anatomically specific connectivity analysis, this pilot study revealed selective and dissociable reconfiguration of thalamocortical and descending control networks following peripheral myofascial stimulation in chronic shoulder pain. The thalamocortical and descending findings support a model in which CSP is maintained by concurrent disruptions in sensory gating and control calibration, while therapeutic response reflects selective recalibration along these axes. Notably, these mechanisms were dissociable at both the group and individual levels, suggesting circuit-specific plasticity in PMS for CSP rather than a single static “pain network.” We emphasize that these conclusions are preliminary and require validation in adequately powered randomized controlled trials.

### 4.1 Thalamocortical gatekeeping of chronic pain

The current work suggests that thalamo-somatosensory coherence may be a key network feature of chronic pain, with both pathophysiological and therapeutic relevance. Baseline dysconnectivity showed a trend toward reduction relative to healthy controls (*p* = 0.054, d = 0.72), and this metric scaled with individual disease burden. While this baseline difference did not reach conventional significance, the medium-to-large effect size and consistent correlations with multiple clinical indices (VAS, ROM, MBI) suggest it may represent a biologically meaningful signal that was underpowered in this small sample. Post-treatment changes in thalamo-somatosensory connectivity were not significant at the group level (*p* = 0.18), but individual variability in plasticity strongly predicted sensory pain relief (*ρ* = −0.73). We interpret this cautiously: the group-level null result does not support a definitive “restoration” mechanism, but the strong individual-level correlation suggests that thalamo-somatosensory plasticity may be a relevant mechanistic target in responders. The observed bidirectional coupling aligns with contemporary refinements of the Gate-Control framework (7, 8). Although the group-level change in thalamo-somatosensory connectivity was not significant, the observed individual-level plasticity raises the possibility that PMS may engage afferent pathways capable of reshaping thalamocortical integration, suggesting a rebalancing of thalamic sensory gating prior to cortical amplification. The use of high-resolution HCP-MMP1.0 parcellation was essential for resolving these subregion-specific effects (e.g., S2 op1-op4) often obscured in coarser atlases.

### 4.2 Descending control recalibration in analgesia

Effective analgesia was associated with an exploratory down-regulation of ACC–brainstem ROI connectivity (*Δ* = −0.166, Wilcoxon *p* = 0.012, d = −0.82) that did not survive Bonferroni correction across the eight *a priori* edges (corrected *p* = 0.098). This finding challenges the simplistic view that pain relief requires “strengthened” descending inhibition. Increasing evidence suggests that various chronic pain states can be characterized by excessive tonic engagement of descending prefrontal circuits that facilitate nociception and related neural activation for the pain experience (33). In this context, the therapeutic effect of PMS-associated decoupling here likely reflects disengagement of an over-engaged pain control system sustaining central sensitization (13) back to a homeostatic baseline, rather than suppression of analgesia. The ACC-brainstem axis may therefore operate as a dynamically adjustable rheostat rather than a binary on-off mechanism.

### 4.3 Dissociable pain dimensions encode distinct network information

This study found a double dissociation: VAS (global unpleasantness) covaried with static baseline connectivity, reflecting cumulative disease burden, whereas PPI (sensory intensity) tracked dynamic connectivity changes, indexing immediate therapeutic gain. This underscores the necessity of distinguishing sensory from affective dimensions in neuroimaging studies (34).

### 4.4 Comparison with standard therapies for chronic shoulder pain

To contextualize our findings, it is important to compare PMS with established standard therapies for CSP, including exercise therapy, physiotherapy, manual therapy, and pharmacological management. Exercise therapy, widely recommended by clinical practice guidelines, has demonstrated moderate efficacy for CSP with effect sizes ranging from 0.3 to 0.6 for pain reduction (35). Physiotherapy interventions (e.g., ultrasound, electrotherapy) show mixed evidence, with some trials reporting significant pain reduction while others find no advantage over placebo (36). Manual therapy, including joint mobilization and soft tissue manipulation, has shown short-term pain relief comparable to exercise but limited long-term benefits (37). Pharmacological management with non-steroidal anti-inflammatory drugs (NSAIDs) provides symptomatic relief but does not address underlying neuroplastic mechanisms (38).

The present study provides preliminary connectomic evidence that PMS may exert analgesic effects through mechanisms distinct from conventional therapies. Specifically, the observed ACC–brainstem decoupling and thalamo-somatosensory plasticity suggest that PMS may target central neuroplastic changes rather than merely modulating peripheral nociception. However, direct comparative neuroimaging studies between PMS and standard therapies are currently unavailable. Whether the observed connectivity changes represent a PMS-specific neural signature or a more general consequence of pain improvement remains an open question. Future studies should include active control groups receiving standard care (e.g., exercise therapy) to dissociate intervention-specific effects from non-specific pain improvement effects. Additionally, the durability of PMS-induced connectivity changes compared to conventional therapies requires longitudinal follow-up beyond the 3-week intervention period.

### 4.5 Specificity of network reorganization

The anatomical specificity of our findings-confined to nociceptive pathways while sparing visual and limbic control networks-is a notable feature. However, we acknowledge that this specificity does not necessarily imply PMS-specificity. Comparable patterns of network reorganization could potentially occur following any effective pain intervention, as pain relief itself may drive recalibration of aberrant connectivity regardless of the intervention modality. For example, cognitive behavioral therapy for chronic pain has been associated with reduced ACC activity and altered descending control network connectivity (39), while exercise therapy has been linked to thalamocortical recalibration in fibromyalgia (40). Without a sham control or active comparator group, we cannot determine whether the observed changes are specific to PMS or represent a general consequence of symptom improvement. We explicitly acknowledge this limitation and recommend that future randomized controlled trials include both sham and active comparator arms to dissociate intervention-specific from non-specific effects.

### 4.6 Relationship between connectivity changes and clinical improvement

An important question is whether the observed network alterations are driven by the PMS intervention itself or by concomitant clinical improvement. To address this, we performed *post-hoc* exploratory analyses controlling for changes in pain intensity (*Δ*VAS) and functional outcomes (*Δ*MBI). When *Δ*VAS was included as a covariate in partial correlation analyses, the correlation between thalamo-somatosensory plasticity and *Δ*PPI was attenuated when controlling for *Δ*VAS (partial *ρ* = −0.51, *p* = 0.09). These exploratory findings suggest that connectivity changes and clinical improvement are intertwined, and we cannot definitively dissociate intervention-specific effects from symptom-driven network plasticity. This is a fundamental limitation of the pre-post design without a control group and underscores the need for randomized controlled trials to establish causal relationships.

### 4.7 Limitations

This study employed a high-resolution, anatomically specific analytical framework, including the HCP-MMP1.0 parcellation scheme to resolve subregional topology, ComBat harmonization to mitigate multi-site technical variance, and permutation-based inference (NBS) to control family-wise error rates. However, when interpreting the results of this study, attention should be paid to several methodological limitations that constrain the strength of our conclusions.

First, the modest sample size (*N* = 12) is a fundamental limitation. As an exploratory pilot study, this sample is insufficient to detect small-to-moderate effects and provides imprecise effect size estimates. While the primary findings were characterized by large effect sizes (|d| > 0.7), suggesting they may represent salient biological signals rather than random noise, the risk of Type II error for null findings and the risk of overestimation for significant findings (“winner's curse”) remain substantial (41). The goal of this work was to produce hypothesis-generating findings, providing preliminary effect size estimates to power future confirmatory trials rather than to establish definitive conclusions. All findings should be interpreted with appropriate caution.

Second, the absence of a sham control group limits definitive causal attribution. We cannot strictly rule out the contribution of non-specific factors such as the placebo effect, regression to the mean, or natural history of recovery. However, the high anatomical specificity of our findings-confined to nociceptive pathways while sparing visual and global control networks-argues against a generic placebo response, which typically manifests as diffuse network alterations (42). Nevertheless, this argument is *post-hoc* and does not substitute for experimental control. Future adequately powered randomized controlled trials with sham control groups are essential to establish causal relationships.

Third, the clinical heterogeneity of the CSP cohort was not stratified. While this approach supports the investigation of central mechanisms shared across diverse shoulder pathologies, it limits the ability to attribute effects to specific peripheral lesions and may increase outcome variability, further reducing statistical power.

Fourth, the healthy control cohort (*n* = 18) serves only as a cross-sectional baseline reference and is not longitudinally assessed. While demographic matching was performed, the sample size is modest for establishing preliminary normative data, and the between-group comparison should be interpreted cautiously.

Fifth, the PMS intervention, while standardized within this study, lacks robust empirical support from large-scale randomized controlled trials. The description provided here represents our institutional protocol and should be validated against independent studies.

Sixth, we did not collect handedness information, which may influence sensorimotor network organization and functional connectivity patterns, particularly given that 67% of CSP participants had right-shoulder involvement and 25% had left-shoulder involvement. Future studies should systematically assess and report handedness. Due to the lack of handedness data, we could not stratify the analysis by hemispheric dominance, which may introduce variability in the sensorimotor connectivity results.

In conclusion, this pilot study provides preliminary connectomic evidence that PMS is associated with the reconfiguration of specific bottom-up gating and top-down control circuits in CSP. The functional connectivity between the anterior cingulate cortex (ACC) and brainstem decreased at the ROI level (*Δ* = −0.166, Wilcoxon *p* = 0.012, d = −0.82; not surviving Bonferroni correction across the eight *a priori* edges, corrected *p* = 0.098), and a negative correlation was identified between thalamic-somatosensory connectivity change and change in present pain intensity (*ρ* = −0.73). These findings highlight the potential of targeting central neural plasticity through peripheral interventions. However, we emphasize that these results are hypothesis-generating: the small sample size, absence of a control group, and inability to dissociate intervention-specific from symptom-driven effects substantially limit causal inference. These circuit-level markers may be leveraged in future works to stratify or monitor response to therapeutic interventions, enabling mechanistically informed personalization of peripheral neuromodulatory therapies. Future adequately powered randomized controlled trials with sham and active comparator arms are warranted to validate these mechanistic targets.

## Data Availability

The raw data supporting the conclusions of this article will be made available by the authors, without undue reservation.
